# A Pipelined Non-Deterministic Finite Automaton-Based String Matching Scheme Using Merged State Transitions in an FPGA

**DOI:** 10.1371/journal.pone.0163535

**Published:** 2016-10-03

**Authors:** HyunJin Kim, Kang-Il Choi

**Affiliations:** 1 School of Electronics and Electrical Engineering, Dankook University, Yongin-si, Republic of Korea; 2 Advanced Communications Research Laboratory, Electronics and Telecommunications Research Institute, Daejeon, Republic of Korea; Nankai University, CHINA

## Abstract

This paper proposes a pipelined non-deterministic finite automaton (NFA)-based string matching scheme using field programmable gate array (FPGA) implementation. The characteristics of the NFA such as shared common prefixes and no failure transitions are considered in the proposed scheme. In the implementation of the automaton-based string matching using an FPGA, each state transition is implemented with a look-up table (LUT) for the combinational logic circuit between registers. In addition, multiple state transitions between stages can be performed in a pipelined fashion. In this paper, it is proposed that multiple one-to-one state transitions, called *merged state transitions*, can be performed with an LUT. By cutting down the number of used LUTs for implementing state transitions, the hardware overhead of combinational logic circuits is greatly reduced in the proposed pipelined NFA-based string matching scheme.

## Introduction

Due to the increasing amount of data and communication speed in computer and network systems, high speed data processing is necessary. In order to extract meaningful information from data, data are searched in order to know whether patterns exist in the middle of the data or not. When a pattern is found, it can be said that the pattern is *matched*. The string that consists of a sequence of characters is the most basic form of patterns. Traditionally, string matching has been an interesting topic for indicating a place where one or several patterns are found. The string matching engine is a specific module that provides the matching information of input data against target patterns. Generally, the string matching engines can be implemented based on the software-based system [1–3]. However, in the era of big data and 100-gigabit communications, the resource in the software-based system cannot be fully focused on the string matching engine. In this case, the software-based string matching engine cannot provide sufficient performance or throughput. For example, in deep packet inspection (DPI), payloads or user data in networking packets should be searched at wire speed. If the throughput does not meet with the wire speed, the performance of network systems can be degraded. Therefore, it is necessary to develop the hardware-based string matching engine.

On the other hand, as the computer and network systems become complex, the number of target patterns increases dramatically. In addition, a set with target patterns can be updated. Therefore, the flexibility in the string matching engine is required to map the updated patterns into the engine. Unlike the software-based string matching engine, the hardware-based string matching engine should adopt a specific device that guarantees the flexibility of the string matching engine. The standard cell-based hardware implementation cannot provide the sufficient flexibility because the hardware structure cannot be changed according to the updated patterns. On the other hand, because FPGA can provide high flexibility using its reconfiguration of logic circuits, the string matching engine can be implemented using an FPGA, which is called the *FPGA-based string matching engine*.

There are several configurable elements in an FPGA. Many kinds of FPGA-based string matching engines using block random access memories (RAMs) have been studied in [4–13], where configurable logic elements have not been used sufficiently. In general, each configurable logic element in an FPGA contains a fixed number of LUTs and flip-flops (FFs). Basically, the configurable logic elements provide the FPGA reconfiguration by changing the contents of LUTs and routing networks in an FPGA. Due to the limited number of logic elements in an FPGA, the FPGA-based string matching engine should have low hardware overhead. In addition, because operating frequency is related to the throughput of the string matching engine, high operating frequency should be achieved.

In an FPGA implementation, as the complexity of a combinational logic circuit increases, great effort for synthesizing and implementing the combinational logic description is required. In addition, operating frequency should be decreased due to the long critical path. On the other hands, an LUT has multiple inputs (generally, from three to six), whereas only one output bit is provided from an LUT [14]. When a combinational logic circuit between registers is simple, several input bits for the LUT cannot be used, where the hardware resource of the unused inputs is wasted. This inefficient resource usage increases hardware overhead in terms of the number of used LUTs. Therefore, the string matching scheme based on FPGA implementation should provide both overall string matching method and its hardware architecture appropriate to the target FPGA, considering hardware complexity, operating frequency, and low hardware overhead.

This paper proposes a pipelined NFA-based string matching scheme for the FPGA-based string matching engine. The characteristics of the NFA such as shared common prefixes and no failure transitions are considered in the implementation of the proposed scheme. In addition, multiple state transitions between stages can be performed in a pipelined fashion. Compared to the implementation of a DFA, when implementing a pipelined NFA, the failure transitions of deterministic finite automaton (DFA) are not needed, so that the number of transitions to be implemented can be small. In addition, because each combinational logic circuit exists between stages separately, each combinational logic circuit can be simplified. Therefore, hardware complexity can be reduced compared to that in the DFA implementation. In the hardware architecture, the pipelined priority encoder is adopted to provide the matching index for the longest matched pattern. In the implementation of the pipelined priority encoder, high operating frequency is achieved by the pipelined encoding, where a hierarchical design with four-input encoders is described. The low hardware complexity of the combinational logic circuits in each stage and pipelined priority encoder can provide high operating frequency. In order to reduce the hardware overhead of combinational logic circuits, it is proposed that multiple one-to-one state transitions are merged and implemented in an LUT. Because the number of used LUTs for implementing state transitions is decreased, the hardware overhead can be reduced. The hardware description for the proposed string matching scheme is automatically generated. Using a commercial tool, the generated hardware description can be compiled and implemented. In the experiments with a state-of-the-art FPGA, the proposed string matching scheme can provide high throughput and low hardware overhead, compared with previous FPGA-based string matching schemes.

This paper is structured as follows: firstly, backgrounds and previous works are explained in detail. Secondly, the proposed string matching scheme and its motivation are described with several illustrations. Finally, experimental results are shown and analyzed in terms of hardware overhead and estimated maximum operating frequency.

## Backgrounds and Previous Works

In this section, the backgrounds of the FPGA-based string matching engine and previous works are explained in detail.

### String Matching using Block RAMs versus Logic Elements

As shown in the previous section, the FPGA-based string matching engines can be implemented using block RAMs or logic elements. When block RAMs are adopted, the operating speed and throughput of the FPGA-based string matching engine depend on the memory access time. Generally, the maximum memory access speed can be kept pace with the maximum operating speed of logic circuits in the state-of-the-art FPGA. However, in order to obtain the next state in one clock cycle, both the memory access and several logic operations are performed, which makes the critical path long. Therefore, due to the low clock frequency caused by the long critical path, the throughput of the FPGA-based string matching using Block RAMs can be lower than that using logic elements.

On the other hand, the time for updating patterns includes the generation of configuration data and pattern mapping in FPGA. When block RAMs are adopted, the automaton-based string matching schemes can be easily implemented by storing next states for each state into the memory. Basically, the next state for a state can be obtained according to input data used as the memory address. In the FPGA-based string matching engines using block RAMs, the hardware description of the string matching engine contains block RAMs. After compiling the hardware description, the string matching engine can be configured. Next, for the pattern mapping, memory contents are generated from target patterns, where memory contents are updated by inputting memory contents into block RAMs. In order to update target patterns, memory contents are re-generated and inputted into block RAMs.

When logic elements are adopted, the hardware description of the string matching engine contains the information of mapped patterns. Therefore, after compiling the hardware description, the generated configuration data include the information of mapped patterns in the form of logic elements. In this case, there is no need to generate memory contents. However, due to the fine granularity of the logic elements, the configuration time can be increased compared to the case using block RAMs. In addition, when target patterns are updated, the compilation of the hardware description should be re-performed.

The maximum capacity of the hardware implementation can be discussed as follows: the size of block RAMs and number of logic elements are limited in an FPGA. For example, the total bits of block RAMs in a Virtex-7 FPGA is 68 Megabits, where the minimum size of a block RAM is 36 Kilobits [15]. On the other hand, 1,955K logic cells can be contained in a Virtex-7 FPGA, in which the logic cell means the logical equivalent of a classic four-input LUT and a FF. For example, in the implementation of the Aho-Corasick algorithm [16] using memory blocks, the next state can be obtained using the current state and input character. When the number of bits in an input character is eight, the number of next state pointers stored in a current state is 256. In addition, the size of a next state pointer can be ⌈*log*_2_ *S*⌉, where *S* is the number of all states. As shown in [17], the Aho-Corasick algorithm requires a large amount of embedded memory for the large rule set such as antivirus applications. Therefore, the implementation of the Aho-Corasick algorithm using an FPGA is not feasible. In [6], the pipelined string matching engine based on the Aho-Corasick algorithm requires 18.1 bits and 0.04 LUT per a character of patterns on average. In the memory-based string matching in [18], the normalized memory requirements for a character of patterns are over 10 bytes. On the other hand, in the implementation of the NFA in [19], a state can be implemented using an LUT. Considering the overall fine granularity and the capacities of block RAMs and logic cells in the state-of-the art FPGA, the string matching engine using logic cells can have larger capacity than that using block RAMs.

Therefore, because the proposed string matching scheme is implemented using configurable logic elements, the string matching engine using configurable logic elements are considered in the later part of this paper, where the string matching schemes using block RAMs are not reviewed.

### Overview of Configurable Logic Elements in an FPGA

An FPGA can provide the flexibility in the string matching engine using configurable logic elements. In general, there are three types of configurable logic elements in an FPGA: LUTs, FFs, and programmable switches. An LUT is small memory cell with several input bits and one output bit [14]. By changing memory contents in each LUT, any logic functions can be implemented. In addition, FFs can be configured for implementing any storage. In an FPGA, several LUTs and FFs are contained in each elementary programmable logic block. For example, a *slice* in Xilinx Virtex-7 FPGA has four six-input LUTs and eight storage elements (FFs) [15]. A *logic element* in Altera Stratix V can have two four-input LUTs and four FFs [20]. There are many homogeneous slices or logic elements in an FPGA, where they are connected using programmable switches. Because an FPGA has a fixed number of slices or logic elements, the numbers of available LUTs and FFs are predetermined, which means that the hardware size for a logic circuit can be limited by the available numbers of LUTs or FFs. In addition, considering the state-of-the-art FPGA structure in [15, 20], the ratio of the number of storage elements to the number of LUTs is increased compared to old-fashioned FPGAs.

### Overview of FPGA-based String Matching Engine

Using the configurable logic elements, several schemes can be applied in the FPGA-based string matching engine. Firstly, TCAM (Ternary Content Addressable Memory) can be emulated. In each row of the emulated TCAM block, a pattern is mapped into comparators. Whereas the commercial TCAM has the fixed number of adopted cells in a row, a row in the emulated TCAM block can vary the number of cells according to the mapped pattern. When the pattern is matched, non-zero matching index is outputted. In order to provide one index for the longest matched pattern, a priority encoder with multiple input indexes should be configured. Because the comparator cells and priority decoder are combinational logic circuits, the ratio of used LUTs to total LUTs is greater than the ratio of used FFs to total FFs. In addition, common prefixes between patterns are not shared. Therefore, resource usage cannot be efficient in the FPGA implementation. Moreover, because the number of comparators for a pattern can increase with the *pattern length* or the number of characters in the pattern, hardware complexity can also increase with the pattern length of each pattern.

In [21], the pipelined comparators are adopted considering the structure of logic cells in an FPGA. By splitting the input data into several bit groups for the LUT, multiple bit-level pipelined comparisons are performed. However, like the TCAM emulation, common prefixes between patterns are not shared explicitly. Even though the pipelined priority encoder is adopted to provide the matching index for the longest matched pattern, the clear structure of the pipelined priority encoder is not shown in [21]. In [22], the pre-decoding scheme is used to share the decoder for the same character between patterns. By sharing the character decoder, the repeated decoding for the same character in each comparator can be avoided, so that the size of combinational logic circuits is reduced. Like [21], the pipelined comparators can be adopted, where the pre-decoding data are inputted into the comparators. However, in [22], common prefixes between patterns are not shared. In addition, the problem of the hardware complexity in the priority decoder is not solved.

In order to share common prefixes between patterns, the automaton-based string matching scheme can be adopted in the FPGA-based string matching engine. In automation-based string matching scheme, states store the information of input data sequence, where state transitions can happen according to the inputted character. In the FPGA-based string matching, states are stored into registers. When the character of a pattern is translated into a state transition, each state transition can be implemented using a combinational logic circuit. The combinational logic circuit outputs the next state according to the inputted character. Between registers for storing the states, the combinational logic circuit for the state transition is placed.

According to the number of current states, automaton-based string matching schemes are categorized into the deterministic finite automaton (DFA) and NFA-based string matching schemes. In the DFA-based string matching scheme, only one state can be the current state. In addition, one state transition can be performed for an inputted character at a time. Therefore, the DFA-based string matching scheme can guarantee the regularity for obtaining the matching index. The DFA-based string matching scheme is simply implemented with both large-sized combinational logic block and register. However, the failure transition for indicating the longest common suffix [16] should be added in each state, which increases hardware complexity. In addition, due to the large size of the combinational logic block, hardware complexity can also be increased.

On the other hand, the NFA-based string matching scheme can have multiple current states and multiple state transitions at a time. Due to the high parallelism using multiple configurable elements, the NFA-based string matching scheme can be implemented using an FPGA. Because multiple current states exist at a time, the information of the longest common suffix is stored into the multiple current states, so that there is no need to indicate the longest common suffix using the failure transition. In addition, because multiple state transitions can be performed at a time, there are many separate combinational logic blocks between registers in the implementation. Therefore, hardware complexity is decreased, compared to that of the DFA-based string matching scheme.

In the NFA-based string matching scheme, in order to perform a state transition, the comparator for recognizing the adequate input data of the state transition is implemented between registers. In an FPGA, the structure of configurable logic elements is fixed. Therefore, if a comparator should be suitably sized considering the structure of the logic elements, the hardware overhead of combinational logic blocks can be reduced.

### Previous NFA-based String Matching Schemes using an FPGA

Because the proposed string matching scheme is the exact string matching, previous NFA-based string matching schemes will be reviewed considering the exact string matching.

In [23], a simple sub-expression can be mapped onto its logic structure. Using the logic structures for a sub-expression, the NFA-based string matching engine can be implemented. However, because the match with each character is mapped onto a comparator, the hardware overhead of combinational logic circuits in an FPGA is not considered.

In the technical approach in [24], both design and implementation processes of an FPGA-based string matching are described. In [24], the balanced utilization between combinational logic and FF resources in the NFA-based string matching is explained. However, there is no systematical or quantitative analysis for the balanced resource utilization.

In the NFA-based string matching in [22, 25], a pre-decoding scheme is used to decode each input character into multiple bits. In [25], considering the structure of each logic element in an FPGA, pipelined state transitions are performed for each pattern in parallel, where each logic element between registers can take the pre-decoding data. However, the sharing of common prefixes between patterns is not considered.

In [19], the NFA-based string matching scheme is implemented in a pipelined fashion. The pre-decoding scheme in [22, 25] is applied in [19]. Conceptually, the string matching scheme in [19] emulates the DFA-based string matching in [16] with the state transitions in different pipelining depths. In addition, common prefixes between patterns are shared. However, because each state transition is implemented using a combinational logic block between registers, the resource usage in terms of used LUTs is inefficient. In addition, due to the long critical path in the priority encoder for providing the matching index, high operating frequency cannot be achieved.

In our previous work in [26], the pipelined NFA-based string matching scheme for emulating the DFA-based string matching is described. When the number of target patterns is small, the hardware overhead for the pre-decoding scheme described in [22, 25] can be great. Therefore, in [26], the string matching scheme does not adopt the pre-decoding scheme. However, like [19], the resource usage and hardware complexity for implementing each state transition and the priority encoder are not considered.

### Pipelined NFA String Matching

Before explaining the proposed pipelined NFA-based string matching scheme, the example of the pipelined NFA string matching scheme based on [19, 22, 25–27] is described. In [27], a memory-based pipelined approach of the Aho-Corasick algorithm is proposed at first time. On the other hand, in [19], the NFA-based string matching scheme is implemented in a pipelined fashion using the logic elements in an FPGA. In the pipelined NFA, like the DFA in [16, 27] and NFA in [19], common prefixes can be shared. Unlike the DFA in [16], the failure transition does not exist in the pipelined NFA.

**Fig 1:**
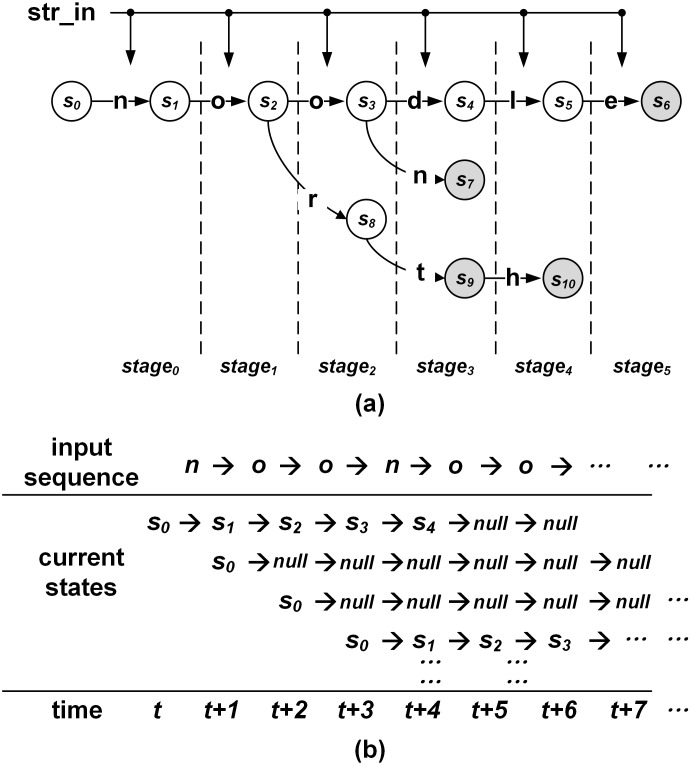
Example of a pipelined NFA: (a) pipelined state transitions for patterns noodle, noon, nort, and north; (b) states in each stage according to an input sequence *noonoo*.

Fig 1(a) shows an example of a pipelined NFA for patterns **noodle, noon, nort**, and **north**. Common prefix **no** is shared between patterns **noodle, noon, nort**, and **north**. Common prefix **noo** is shared between patterns **noodle** and **noon**. Common prefix **nort** is shared between patterns **nort** and **north**. The circle and number in the circle mean the state and index of the state at a stage, respectively. The circle with gray color represents the output state, where a pattern can be matched. The normal arrow means a state transition from a state towards another state. When the input is the character on the normal arrow, the state transition is performed. The vertical dotted line shows the pipeline stage according to the distance from the initial state *s*_0_. In a stage of the pipelined NFA, only one state can be the current state. If the maximum pattern length is *L*, there are *L* pipeline stages (*stage*_0_ − *stage*_*L*−1_) and *L* current states. When a character is inputted, state transitions can be performed from *stage*_*i*_ to *stage*_*i*+1_ in parallel. If there is no input related to any valid state transitions in *stage*_*i*_, the next state in *stage*_*i*+1_ can be a null state.

Fig 1(b) describes states in each stage according to an input sequence *noonoo*. When each character is inputted, the state in each stage is determined in a pipelined fashion. Because there is no failure transition, if the current state is a null state, the next state can also be another null state. In Fig 1(b), when a character sequence *noonoo* is inputted, the states in *stage*_4_ and *stage*_5_ at *time* = *t*+5 and *time* = *t*+6 are null states, which means that there are no matched patterns with lengths of 5 and 6 for the input sequence *noonoo*. Because the current state exists in each stage, there can be multiple output states. When the index of the longest matched pattern should be provided, the output state in *stage*_*j*_ has priority over the output state in *stage*_*i*_ when *i* < *j*.

**Fig 2:**
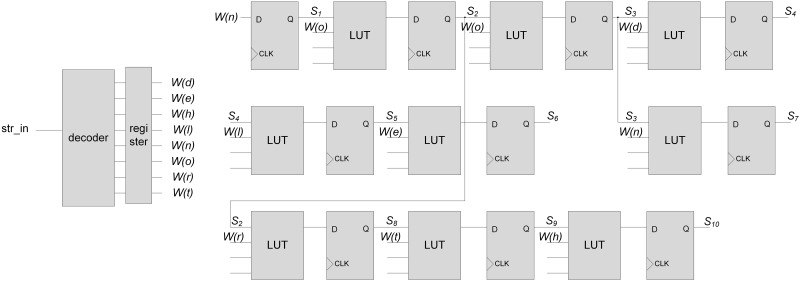
Circuit diagram of the pipelined NFA in Fig 1.

Fig 2 shows a circuit diagram of the pipelined NFA in Fig 1. Like [22, 25], the character decoder is implemented. The decoded output bits are inputted into the comparators in LUTs for performing state transitions, where bit *W*(*c*) can be one for an input character *c*.

By adopting the one-hot encoding, the values stored in FFs for a pipelined stage indicate the current state in a stage. For a stage, only one FF can have the output value of one (or true); other FFs have the output values of zero (or false). By ANDing the decoded output bit and output value from each FF, the input value of each FF for the next stage is determined. Considering the example in Fig 1, the number of decoded output bits for each state can vary due to the different numbers of state transitions from each state.

When an output state is reached, the identification number of the matched pattern should be provided. When the value of a state is one, the state can be a current state. Because multiple states can be current states in the pipelined NFA, there are multiple output states at a time. In order to provide the identification number for the longest matched pattern, a priority encoder should be adopted. Let us assume that the matching indexes 1, 2, 3, and 4 are assigned for states *s*_6_, *s*_7_, *s*_9_, and *s*_10_ for the pipelined NFA in Fig 1(a). When *i* < *j*, the pattern length for the output state in *stage*_*j*_ is longer than that for the output state in *stage*_*i*_. Therefore, the output state in *stage*_*j*_ has priority over that for the output state in *stage*_*i*_. For example, state *s*_6_ has priority over states *s*_7_, *s*_9_, and *s*_10_, where the matching index of 1 is outputted for the longest matched pattern.

## Proposed String Matching Scheme

In this section, the motivation and detail description of the proposed FPGA-based string matching scheme are provided. In this section, it is assumed that the proposed string matching scheme takes one character as an input at a time. Even though the proposed string matching scheme can be extended into the string matching with multiple input characters, the hardware complexity can increase with the number of inputted characters. In addition, as shown the parallel string matching scheme in [28, 29], multiple input chunks or sequences of characters can be provided, where each chunk can be inputted into each homogeneous string matching module. However, in order to show the proposed idea clearly, the proposed string matching scheme is explained for the case with one inputted character at a time.

### Motivation

In the FPGA-based string matching engine, states and state transitions are implemented using FFs and LUTs, respectively. In the previous NFA-based string matching scheme in an FPGA, each state transition between states can be implemented with an LUT. If the number of used input bits in an LUT is smaller than the maximum number of input bits available in the LUT, the resource for implementing the combinational logic circuits in an FPGA is wasted. In Fig 2, each two-input AND gate is implemented using an LUT, where the decoded output bit of a character and output bit from an FF for the current state are ANDed. Whereas the maximum number of input bits in an LUT is four, the number of used input bits in each LUT is only two. Therefore, we propose a new NFA-based string matching scheme that increases the efficiency in the resource usage of combinational logic circuits for implementing state transitions.

On the other hand, as the number of patterns mapped in an NFA increases, the hardware complexity in the priority decoder also increases. Due to the increased hardware complexity with the number of mapped patterns, the critical path could exist in the priority encoder. Therefore, by adding pipeline registers, a pipelined priority encoder can be adopted to shorten the critical path. Even though the string matching engine in [21] can adopt a pipelined priority encoder, the detail structure of adopting the pipelined priority encoder for the proposed FPGA-based string matching will be described.

### Merged State Transitions in Pipelined NFA String Matching

Considering the structure of logic elements in an FPGA mentioned above, we propose a new idea called *merged state transitions* that can enhance the efficiency of resource usage in the combinational logic circuits. In order to describe the merged state transitions, the pipelined NFA in Fig 1(a) is modified.

For finding the candidates of state transitions to be merged, the state transitions to be merged should meet all of the following conditions:

1. The state transitions to be merged are related to the subpattern or character sequence of a target pattern.
2. When a state transition *st*_*j*_ goes towards state *s*_*i*_, if only one valid state transition *st*_*k*_ comes from state *s*_*i*_, the state transitions *st*_*j*_ and *st*_*k*_ can be merged.
3. The state transition from an output state cannot be merged with the state transition towards the output state.
4. A state transition can be merged only once.

The purpose of the conditions mentioned above is explained as follows: firstly, the state transitions for the match with a target pattern are performed in sequence. Therefore, the state transitions to be merged should be related to the subpattern of the target pattern.

On the other hand, a subpattern with the sequence of more than one characters is translated into a set of merged state transitions. Let us assume that the state transition towards state *s*_*i*_ are shared between multiple target patterns. When state *s*_*i*_ has more than one state transition towards other states, not all state transitions towards other states can be shared between the target patterns. Therefore, if the state transition towards state *s*_*i*_ is merged with one state transition from state *s*_*i*_, the merged state transitions cannot be shared. In this case, the subpattern of the merged state transitions for a target pattern is not always the subpattern for another target pattern. Therefore, in order to share the state transition towards a shared state *s*_*i*_, the state transition from state *s*_*i*_ cannot be merged with the state transition towards state *s*_*i*_.

If the state transition from an output state is performed, the output state has been reached. On the other hand, for the merged state transitions, a sequence of characters is compared with a subpattern at a time. When the state transition from an output state is merged with any other state transition towards the output state, there is no way to know whether the output state has been reached or not. Therefore, the state transition from an output state cannot be merged with the state transition towards the output state.

If a state transition *st*_*j*_ is merged with other state transitions, the state transition *st*_*j*_ can be the element of a set for comparing a sequence of characters at a time. If the state transition is merged more than once, the state transition can be the element of another set. In this case, the state transition can be performed several times. Therefore, the condition that a state transition should be merged only once should be met.

**Fig 3:**
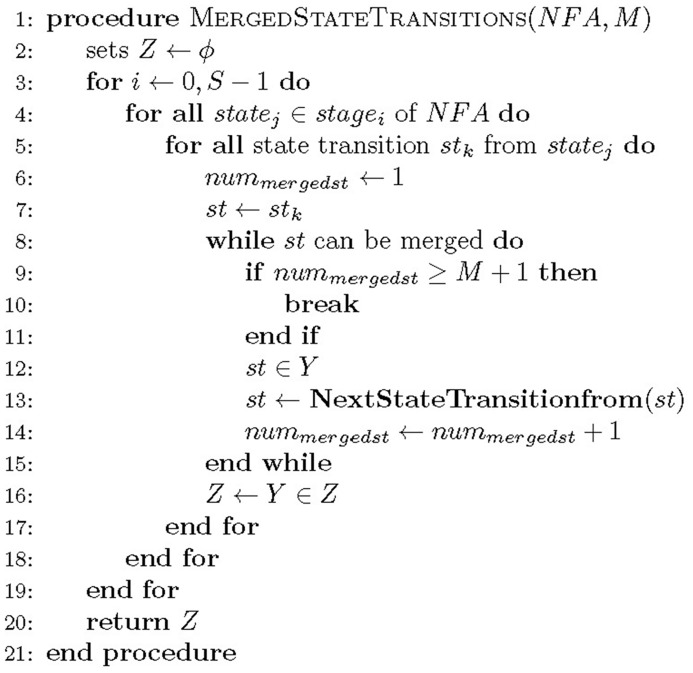
Pseudo code for extracting sets of state transitions to be merged.

Fig 3 describes a procedure that extracts sets of state transitions to be merged. The procedure gets the information of a pipelined NFA (*NFA*) and the maximum number of merged state transitions for a state (*M*) as inputs. Breadth-first search (BFS) is applied to search all states (*state*_0_ − *state*_*S*−1_) from *stage*_0_. In stage *stage*_*i*_, a state transition *st*_*k*_ of state *state*_*j*_ is stored into a temporary variable *st*. If state transition *st* can be merged and the number of state transition to be merged *num*_*mergedst*_ is smaller than *M* + 1, state transition *st* can be an element of set *Y*, which contains the state transitions to be merged. The next state transition is obtained with *st* by calling procedure **NextStateTransitionfrom**. Then, variable *st* is replaced with the next state transition. This process will be iterated until the maximum number of state transitions can be merged. It is noted that the minimum number of state transitions included in set *Y* can be one. After finishing **while** loop, the final set *Y* becomes a new element in *Z*. Finally, all sets of state transitions to be merged (*Z*) are returned.

**Fig 4:**
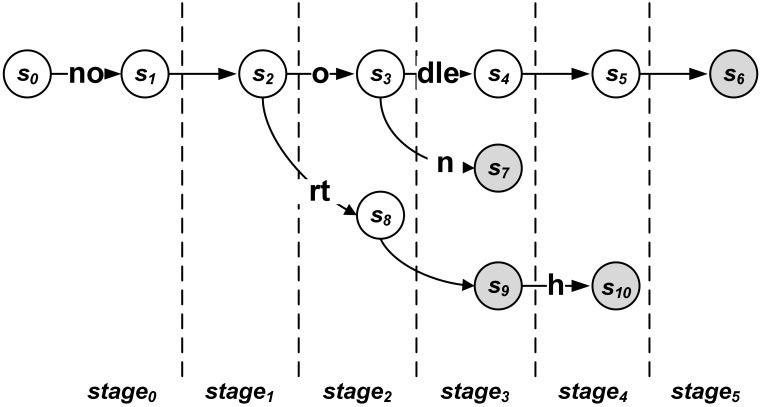
Example of the pipelined NFA using merged state transitions.

Fig 4 describes an example of the pipelined NFA using merged state transitions. In Fig 4, subpatterns **dle, no**, and **rt** are adopted to merge state transitions. Compared to Fig 1(a), the state transition from state *s*_0_ is merged with the state transition from state *s*_1_ because state *s*_1_ has only one valid state transition towards state *s*_2_. The state transition from state *s*_9_ is not merged because state *s*_9_ is the output state.

**Fig 5:**
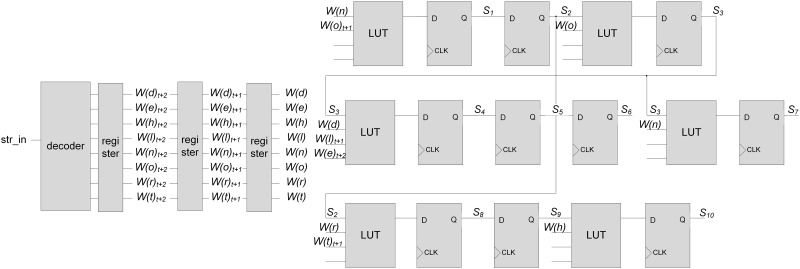
Circuit diagram of the pipelined NFA using merged state transitions in Fig 4.

Fig 5 shows a circuit diagram of the implementation for the pipelined NFA using merged state transitions in Fig 4. For the four-input LUT in an FPGA, a maximum of three merged state transitions can be inputted with a value of previous state. In order to support merged state transitions at a time, the input character is decoded, and then the decoded output bits are shifted. In the left part of Fig 5, registers are adopted for shifting the decoded output bits. The outputs from the registers are inputted into the comparator of merged state transitions. For example, if three state transitions are merged, three decoded output bits are inputted for the comparison with three input characters at a time. Therefore, compared to the decoder in Fig 2, two additional registers are adopted, where decoding bit *W*(*c*)_*t*+*i*_ can be one for an input character *c* at *time* = *t* + *i*. When *i* is zero, the subscript *t* + *i* is removed for clarity. The comparators for the merged state transitions are implemented using LUTs. Compared to the circuit diagram in Fig 2, multiple decoding bits are inputted in several LUTs in Fig 5, where the required number of LUTs is reduced from nine to six.

When state transitions are merged, the state information can be shifted using the chain of FFs. Because the state information is stored in FFs each time, the number of FFs for storing the state information does not change, compared to that shown in Fig 2. On the other hand, several registers for shifting the output bits from the character decoder are required. When the maximum number of merged state transitions for a state is *M*, the maximum number of FFs required for shifting the decoded output bits can be 256 × (*M* − 1) for ASCII character inputs. Actually, because all ASCII characters are not shown in target patterns, the increased number of FFs can be smaller than the maximum number. When merging state transitions, whereas the number of used LUTs can be reduced, the number of used FFs can be increased. Therefore, the idea with the merged state transitions reduces the number of used LUTs greatly with slightly increased number of used FFs.

### Identification of Matched Patterns with Pipelined Priority Encoder

As shown in the previous section, the identification number of the longest matched pattern is provided with the priority encoder. In order to decrease hardware complexity, a pipelined priority encoder can be adopted, so that high operating frequency is achieved.

On the other hand, according to the target patterns, a specific priority encoder can be implemented. For example, when the lengths of several patterns are equal, the output states for the patterns are located in a stage. Because only one state in each stage can be the current state, the hardware complexity of implementing the priority encoder can be reduced. In this case, in order to show the identification of the stage for the matched longest pattern, the index of the stage is provided using the priority encoder. In addition, the encoder for each stage is required for providing the index of the output state of the matched pattern. Therefore, the identification number of the pattern for a state can be the combination with the index of the state in a stage and the index of the stage. In this case, the size of the identification number depends on the distribution of pattern lengths.

**Fig 6:**
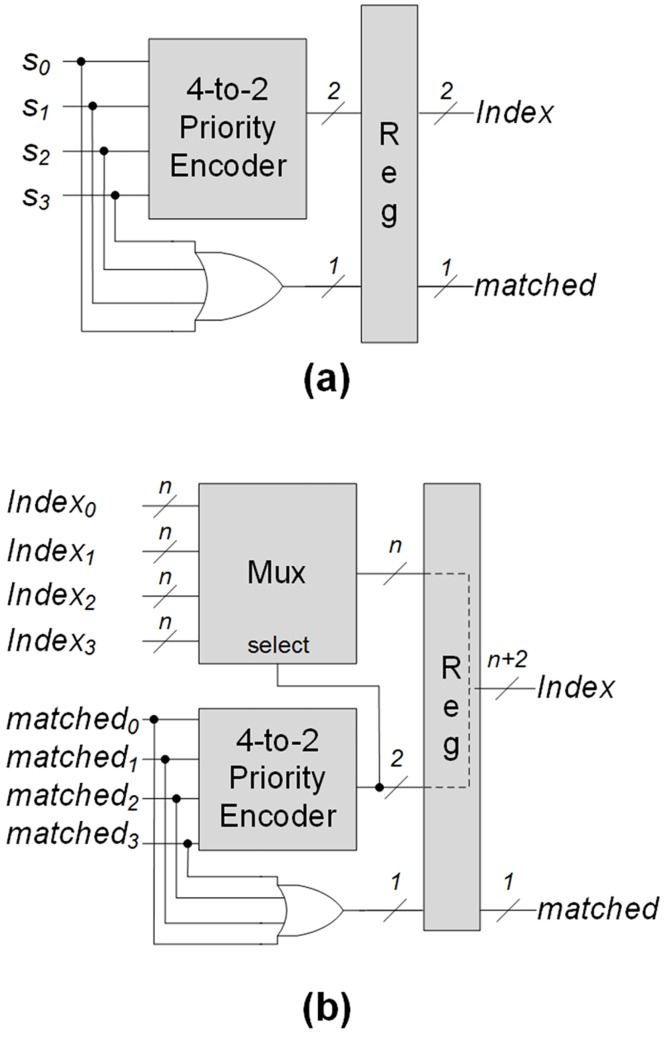
Blocks of pipelined priority encoder: (a) an unit block for getting information of output states; (b) a block for the stage with four *n*-bit indexes and matched signals.

In our implementation of the pipelined priority encoder, instead of designing the specific pipelined priority encoder according to different set of target patterns, a hierarchical design of the pipelined priority encoder is adopted as follows: firstly, a unit block of the pipelined priority encoder is shown in Fig 6(a), where bits *s*_0_, *s*_1_, *s*_2_, and *s*_3_ are the information of four output states. Multiple unit blocks are adopted in the first pipeline stage. If the number of target patterns is *x*, the number of unit blocks can be $\left\lceil \frac{x}{4}\right\rceil$. If an output state *s*_*i*_ is reached, signal *s*_*i*_ in Fig 6(a) can be true; otherwise, signal *s*_*i*_ is false. If *i* < *j*, output state *s*_*i*_ has priority over output state *s*_*j*_. The priority encoder in Fig 6(a) is the combinational logic circuit with four-input bits. A four-input OR gate, which is used to generate an output signal *matched* with signals *s*_0_, *s*_1_, *s*_2_, and *s*_3_. Signal *matched* indicates whether there are any matched patterns or not. There are two reasons why four bits are inputted; firstly, because each LUT has one output bit, four input bits can be encoded with two output bits with two LUTs. Secondly, considering the number of inputs in an LUT of commercial FPGAs, four input bits can be sufficient to generate signal *matched*.

Except for the first stage, multiple-bit indexes from a previous stage are inputted. Fig 6(b) describes a block for the stage with four *n*-bit indexes and *matched*_*i*_ signals from the previous stage. When the number of target patterns is *x*, ⌈*log*_4_ *x*⌉ stages are required. Output signal *matched* indicates whether there are any matched patterns by ORing input signals *matched*s from the previous stage. Using the 4-to-2 priority encoder, two-bit selection signal is provided for the *n*-bit multiplexor, which can transmit the matching index with the highest priority from the previous stage. The matching index of this stage is provided by concatenating the two bits from the 4-to-2 priority encoder (high-order bits) and *n* bits from the multiplexor (low-order bits).

**Fig 7:**
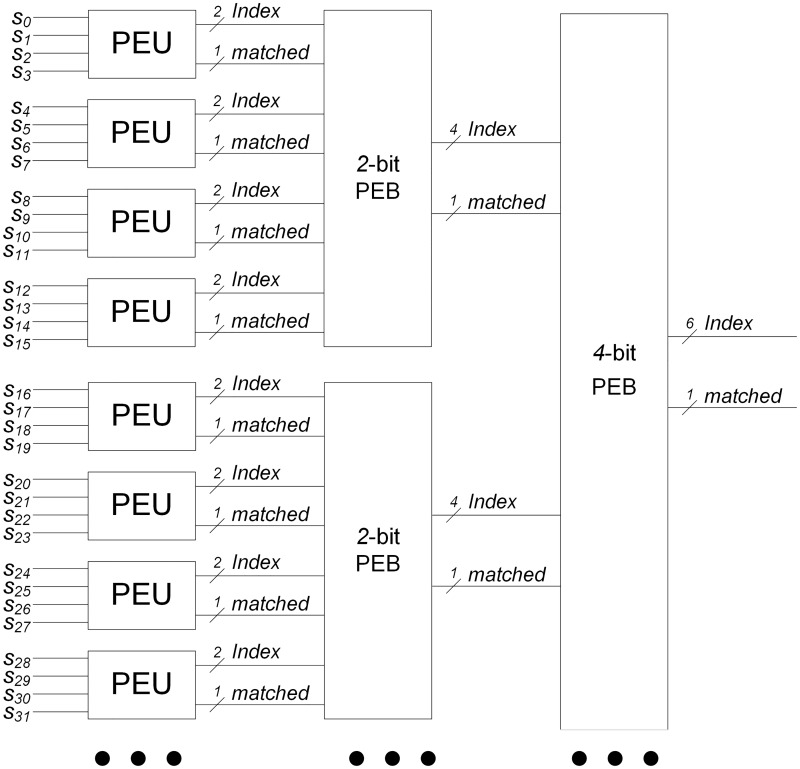
An example of pipelined priority encoder.

Based on the detail explanation of Fig 6 mentioned above, Fig 7 shows a diagram of the pipelined priority encoder. In Figs 7 and 6(a) and 6(b) are denoted as *PEU* (priority encoder unit) and *n*-bit *PEB* (priority encoder block), respectively. Even though the information of 32 output states are shown in Fig 7, the hierarchical and regular design can be possible. In addition, the size of the identification number for *n* patterns can be ⌈*log*_2_ *n*⌉. For 32 output states, two 2-bit PEBs provide two 4-bit indexes; the MSB (most significant bit) of the 6-bit index of the 4-bit PEB can be logical zero. Therefore, the 5 leftmost bits can be outputted for identifying matches with 32 patterns in Fig 7.

## NFA Construction and Hardware Implementation

There are several issues for constructing NFA and implementing hardware, which will be discussed in the following subsections.

### NFA Construction

**Fig 8:**
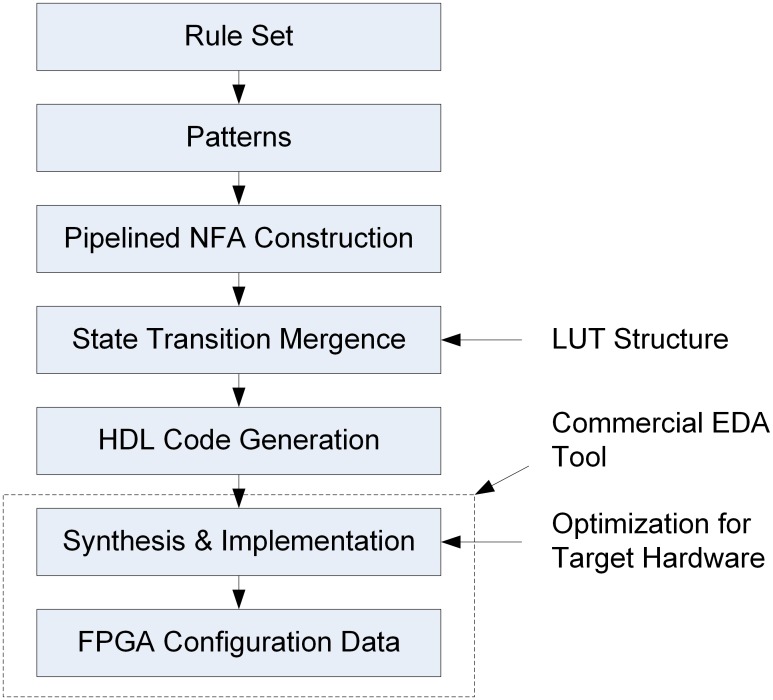
Flow to obtain FPGA configuration data from a rule set.

Fig 8 shows the flow to obtain FPGA configuration data from a rule set. In order to construct a pipelined NFA, patterns are extracted from a rule set. By mapping the patterns onto the generated pipelined NFA, the pipelined NFA construction is performed. Each character in a pattern is mapped onto a valid state transition, which is similar to the goto function of the Aho-Corasick algorithm. On the other hand, the pipelined NFA does not adopt the failure function. Based on the constructed pipelined NFA, the state transitions are merged, where the LUT structure in a target FPGA is considered. After merging the state transitions, a register-transfer level (RTL) HDL code is generated, where the generated code is synthesizable in the commercial EDA tool. After synthesis and implementation with the generated HDL code, the FPGA configuration data is obtained.

### Hardware Implementation and Updatability

In the synthesis and implementation step, the resource usage can be reported. When the resource usage is not under the resource limitation of the target FPGA, it is concluded that additional FPGA should be adopted. In addition, the HDL code for constructing a new NFA is required.

On the other hand, when a target rule set is updated, the generated HDL code should be changed, where the code is synthesized and implemented. Because the configuration time of an FPGA can be great, several suitable solutions are required. In this case, if a redundant FPGA is used, there is no need to stop the string matching engine when updating the rule set. While configuring a redundant FPGA for the new rule set, other FPGA is performing the string matching for an old rule set. After finishing the configuration of the redundant FPGA, the redundant FPGA is replaced by the FPGA that runs the string matching. Then, the newly configured device can run. At the same time, the old FPGA can be a redundant device. In addition, the partial reconfiguration can be helpful to solve the problem of updated rule sets. Now, major FPGA vendors support the partial reconfiguration, as shown in [30, 31]. By adopting the partial reconfiguration flow, the functionality of the changed patterns can be changed on the fly. Considering the solutions for updated rule sets mentioned above, it seems that the FPGA-based string matching can be realistic in the commercial FPGAs.

## Experimental Results

### Experimental Environments

In order to evaluate the proposed FPGA-based string matching scheme, a program was made using the C++ library and Boost graph library (BGL) [32]. A RTL HDL code was generated by our in-house program, considering target patterns and string matching scheme. The program ran on a Linux machine with Centos 5.11 operating system, Intel(R) Xeon E5620 CPU @2.4 GHz with 8 threads, and 6-Gigabyte physical memory.

Eight different rule sets were adopted from Snort [33] v2.8 rules. Table 1 enumerates several characteristics of rule sets. Considering *σ*, pattern lengths in a rule set were widely spread. The distributions of pattern lengths for rule sets could be different from each other. Therefore, it was concluded that the adopted rule sets were sufficient for evaluating the proposed string matching scheme.

**Table 1:** Characteristics of rule sets with target patterns.

rule set	num(patterns)^1^	num(bytes)^2^	max(*l*)^3^	avg(*l*)^4^	*σ* ^5^
backdoor	955	8875	94	9.3	7.5
chat	49	431	38	8.8	8.5
deleted	615	7399	72	12.0	11.0
exploit	243	1906	109	7.8	9.2
oracle	337	10783	53	32.0	12.6
policy	114	1154	112	10.1	12.7
spyware	2299	26103	94	11.4	8.1
web-client	1657	67527	92	40.8	22.8

^1^Number of patterns.

^2^Total number of characters of target patterns in rule set.

^3^Maximum pattern length.

^4^Average pattern length.

^5^Standard deviation of pattern lengths.

In the experiments, our target FPGA was Xilinx xc7vx690t-2-ffg1157 [15], which had 866,400 slice registers and 433,200 LUTs with six input bits. The generated HDL code was synthesized using XST in 64-bit ISE 14.4 of Xilinx [34]. The operating system was 64-bit Microsoft Windows 7 that ran on Intel(R) I7-5960x CPU @3.0 GHz with 16 threads, and 32-Gigabyte physical memory.

### Experimental Data and Discussion

**Fig 9:**
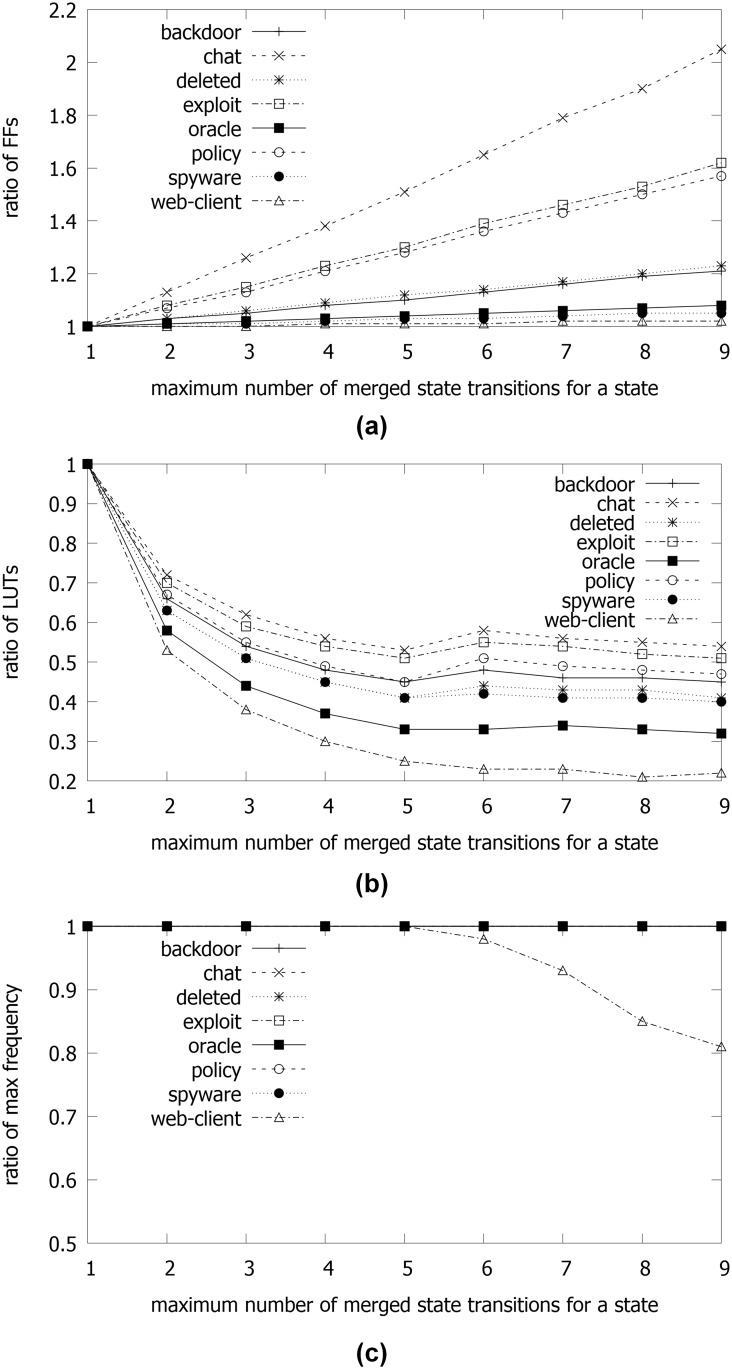
Comparisons by sweeping the maximum number of merged state transitions for a state *M*: (a) ratios of used FFs to that when *M* = 1; (b) ratios of used LUTs to that when *M* = 1; (c) ratio of the maximum operating frequency *F* to that when *M* = 1.

In order to calculate both hardware overhead and maximum operating frequency according to the maximum number of merged state transitions for a state denoted as *M*, evaluations were performed by sweeping *M*. Fig 9 shows the ratios of numbers of FFs, LUTs, and the maximum operating frequency *F* to those when *M* = 1. The number of FFs for shifting the decoded output bits of character inputs increased with *M* in all rule sets. In the rule sets with small number of target patterns, the ratio of FFs for shifting the decoded output bits to those for implementing states in the pipelined NFA can be great. Therefore, for the rule sets with small number of target patterns such as *chat*, *exploit*, and *policy*, the ratios increased sharply with *M*, as shown in Fig 9(a).

On the other hand, the required numbers of LUTs decreased until *M* = 5. Considering the structure of a pipelined NFA like a tree, the ratio of state transitions to be merged can be great in the rule sets with the long average pattern length such as *oracle* and *web-client*. In the target FPGA with six-input LUTs, the maximum number of merged state transitions for a state can be five for an LUT. In this implementation, five decoded output bits of character inputs for merged state transitions and the value of a state can be ANDed in an LUT. When *M* = 6, the ratios of LUTs in several rules were increased except for *web-client*. In *web-client*, due to the repeated subpatterns with **0…0** in each pattern, it was analyzed that the combinational logic circuit of the comparators for performing merged state transitions was optimized for low hardware overhead. Therefore, it was concluded that the maximum number of inputs in an LUT was related to the hardware overhead by merging state transitions. In addition, it was expected that the amount of reduced hardware overhead can depend on a set of target patterns. In summary, compared to the cases without merging state transitions (*M* = 1), the required number of LUTs decreased by 75.1%-46.9% when *M* = 5.

Maximum operating frequencies were not changed until *M* = 5 in all rule sets. From *M* = 6, the maximum operating frequency of *web-client* decreased. After analyzing the synthesis report for *web-client*, large routing delay was found in the circuit that transmitted shifted decoded output bits into the comparator for merged state transitions. Due to the large pattern length in *web-client*, it was concluded that the routing complexity for large *M* can be great. Considering the analysis mentioned above, there was a threshold point of *M*, which was related to the structure of an LUT. Therefore, the experimental data with *M* = 5 for the target FPGA will be adopted in the later part of this section.

Table 2 shows several evaluation data of the proposed string matching scheme. A lot of characters were in shared common prefixes of each rule set, where the ratios of characters in shared common prefixes to all characters for a rule set were ranged from 54.6% to 9.3%. In order to compare with the previous pipelined NFA, the number of *moved* state transitions to be merged was computed. In these computations, when the number of merged state transitions was *m*, *m* − 1 state transitions were moved; one state transition can be unmoved in the same stage, compared to the previous pipelined NFA. As shown in Table 2, 78.5%-69.6% of state transitions were moved to be merged. Therefore, it was expected that there were many state transitions to be merged for reducing hardware overhead.

**Table 2:** Evaluation data with proposed string matching scheme.

rule set	#shared^1^	#merged^2^	*r*(shared)^3^	*r*(merged)^4^	*t*(patterns)^5^	*t*(XST)^6^
*backdoor*	2308	4629	26.0%	70.5%	0.21sec	40.2sec
*chat*	82	249	19.0%	71.6%	0.01sec	20.3sec
*deleted*	1845	4019	24.9%	72.4%	0.28sec	37.8sec
*exploit*	321	1103	16.8%	69.6%	0.08sec	26.5sec
*oracle*	5888	3689	54.6%	75.4%	0.15sec	42.7sec
*policy*	118	754	10.2%	72.9%	0.02sec	24.5sec
*spyware*	8006	12860	30.7%	71.1%	0.91sec	114.6sec
*web-client*	6303	48055	9.3%	78.5%	1.74sec	347.6sec

^1^Number of characters in shared common prefixes.

^2^Number of moved state transitions to be merged.

^3^Ratio of characters in shared common prefixes to all characters.

^4^Ratio of moved state transitions to all state transitions.

^5^Time required for generating the HDL code.

^6^Time required for completing synthesis using XST, which does not include the implementation (placement and routing) time.

On the other hand, the times required for generating a HDL code and synthesizing the code were estimated for each rule set. Assuming that the number of target patterns was *N*, the time complexity of constructing an NFA and extracting merged state transitions can be O(*N*). Considering data in Table 2 and the time complexity, it was expected that the time required for generating an HDL code was not be great for any rule set. In addition, it was analyzed that the time required for synthesis was not great for the adopted rule sets.

Table 3 summarizes the comparisons with other FPGA-based string matching schemes in terms of numbers of LUTs and FFs. In addition, the maximum operating frequency *F* of each string matching scheme is shown. Several data denoted as *N/A* in *pipe_cam*, *dfa*, and *dfa_onehot* were not computed after 24-hour synthesis using the commercial tool due to their high hardware complexity.

**Table 3:** Comparisons with other FPGA-based string matching schemes in terms of numbers of FFs & LUTs and maximum operating frequency *F*.

	*cam* ^1^		*pipe*_cam^2^		*dfa* ^3^		*dfa_onehot* ^4^		*ppfac* ^5^		*pipe_nfa_cd* ^6^		*pipe_nfa_cd_ppe* ^7^		*proposed*	
rule set	#FFs	#LUTs	#FFs	#LUTs	#FFs	#LUTs	#FFs	#LUTs	#FFs	#LUTs	#FFs	#LUTs	#FFs	#LUTs	#FFs	#LUTs
*backdoor*	1,717	5,610	3,431	7,244	N/A	N/A	6,584	18,936	432	13,788	6,811	8,863	7,979	8,145	8,812	3,640
*chat*	359	434	1,119	728	39	1,808	362	500	143	547	424	468	485	473	731	251
*deleted*	1,202	4,555	7,545	6,024	32	72,620	5,571	11,756	459	10,458	5,778	7,059	6,539	6,618	7,293	2,708
*exploit*	1,123	1,877	3,678	2,344	39	15,843	1,600	3,316	305	2,968	1,773	2,154	2,070	2,055	2,694	1,043
*oracle*	770	2,790	5,141	4,515	39	27,451	4,911	6,846	441	8,053	4,964	5,575	5,392	5,463	5,605	1,800
*policy*	1,097	1,163	2,760	1,590	71	18,111	1,050	1,710	259	1,728	1,149	1,299	1,295	1,278	1,661	573
*spyware*	3,063	11,259	18,700	14,956	N/A	N/A	N/A	N/A	558	36,785	18,262	23,623	21,087	21,711	21,618	8,930
*web-client*	2,406	14,163	N/A	N/A	N/A	N/A	N/A	N/A	1,005	58,834	61,421	64,819	63,462	63,846	64,105	15,904
rule set	*F*(MHz)		*F*(MHz)		*F*(MHz)		*F*(MHz)		*F*(MHz)		*F*(MHz)		*F*(MHz)		*F*(MHz)	
*backdoor*	232.6		679.1		N/A		193.7		194.8		226.2		679.1		679.1	
*chat*	340.0		695.7		189.9		384.7		352.0		405.1		695.7		695.7	
*deleted*	250.7		687.0		130.8		209.8		162.0		246.6		687.0		687.0	
*exploit*	288.8		689.5		165.0		248.9		238.6		297.3		689.5		689.5	
*oracle*	288.8		687.0		155.7		248.2		188.9		268.6		687.0		687.0	
*policy*	335.3		695.3		107.8		298.7		251.1		362.7		695.3		695.3	
*spyware*	199.6		671.4		N/A		N/A		169.8		193.6		671.4		671.4	
*web-client*	220.4		N/A		N/A		N/A		181.8		220.0		676.8		676.8	

^1^TCAM emulation.

^2^Pipelined TCAM emulation with a pipelined priority encoder in [21].

^3^DFA-based string matching in [16] with binary state encoding.

^4^DFA-based string matching in [16] with one-hot state encoding.

^5^Pipelined NFA-based string matching in [26] without pre-decoding scheme, pipelined priority encoder, and merged state transitions.

^6^Pipelined NFA-based string matching in [19] with pre-decoding scheme and without pipelined priority encoder &merged state transitions.

^7^Pipelined NFA-based string matching with pre-decoding scheme & pipelined priority encoder and without merged state transitions.

Especially, in *web-client*, due to the repeated subpatterns mentioned above, it was expected that the required number of LUTs was optimized by the synthesis tool. Therefore, compared to the case in *cam*, the required number of LUTs was not decreased only for *web-client*. Except for the case, the required numbers of LUTs were smallest in *proposed*, compared to other schemes. Especially, compared to the cases of the pipelined NFA string matching scheme in [19], the required numbers of LUTs were decreased by 75.5%-46.4%.

On the other hand, it was noted that the required numbers of FFs were increased for shifting the decoded output bits of character inputs and implementing the pipelined priority encoder. In addition, considering several characteristics in Table 1 and experimental data in Table 3, it was concluded that the ratio of the increased FFs was negligible as the numbers of states and state transitions were great. The comparison of data between *pipe_nfa_cd* and *pipe_nfa_cd_ppe* gave the information of the increased hardware overhead due to the pipelined priority encoder. With this comparison and data in Fig 9(a), it was analyzed that most additional FFs was caused by the implementation of the pipelined priority encoder for large rule sets such as *spyware* and *web-client*.

In comparisons in terms of *F*, the experimental data for *pipe_cam*, *pipe_nfa_cd_ppe*, and *proposed* with the pipelined priority encoder showed high *F*s. These three schemes with high *F*s adopted the same pipelined priority encoder for each rule set. Because critical paths existed in the pipelined priority encoder, *pipe_cam*, *pipe_nfa_cd_ppe*, and *proposed* showed the same *F*s in Table 3. Even though *cam*, *dfa*, and *ppfac* required the small numbers of FFs, *F*s were not high. In addition, the proposed pipelined NFA-based string matching scheme provided high *F*s with small deviation, where the throughput for one character input at a time can be reached up to 5.56 Gigabits per second (Gbps) -5.37 Gbps.

Table 4 summarizes the comparisons with other FPGA-based string matching schemes in terms of numbers of used LUT & FF pairs and fully used LUT & FF pairs. In order to know the design density and distribution, the data about the logic distribution are added. As shown in [35], the LUT &FF pairs can be a cluster to take advantage of the design locality in an FPGA. If logic clusters contained insufficient logic resources, the amount of inter-cluster routing resources needed for routing will be great. Therefore, the complexity of wiring and interconnections can be predicted. In Table 4, the ratios of the number of fully used LUT & FF pairs to the number of used LUT & FF pairs in *cam*, *aho*, and *ppfac* were very low, which could increase the routing resources between clusters. The main reason of the low ratios was because the number of FFs was much smaller than the number of LUTs. In *proposed*, the ratios of the number of fully used LUT & FF pairs to the number of used LUT & FF pairs were low, compared to the cases of *pipe_nfa_cd* and *pipe_nfa_cd_ppe*. Considering the low ratios of *proposed*, the additional routing resources between clusters in *proposed* can be required. Due to the largely decreased number of used LUTs, the ratios can be lowered. Compared to *pipe_nfa_cd* and *pipe_nfa_cd_ppe*, because the ratios of increased FFs in small rule sets such as *chat* and *policy* were somewhat high, the ratios of the number of fully used LUT & FF pairs to the number of used LUT & FF pairs can be proportionally lowered. However, for large rule sets such as *spyware* and *web-client*, due to the low ratio of increased FFs, it was expected that the additional routing resources between clusters could be small.

**Table 4:** Comparisons with other FPGA-based string matching schemes in terms of numbers of used LUT & FF pairs and fully used LUT & FF pairs.

	*cam* ^1^		*pipe*_cam^2^		*dfa* ^3^		*dfa_onehot* ^4^		*ppfac* ^5^		*pipe_nfa_cd* ^6^		*pipe_nfa_cd_ppe* ^7^		*proposed*	
rule set	#Pairs^8^	#Fulls ^9^	#Pairs	#Fulls	#Pairs	#Fulls	#Pairs	#Fulls	#Pairs	#Fulls	#Pairs	#Fulls	#Pairs	#Fulls	#Pairs	#Fulls
*backdoor*	6,404	923	9,703	6,972	N/A	N/A	18,942	6,578	13,851	369	9,172	6,502	8,343	7,781	9,295	3,157
*chat*	732	61	1,131	716	1,831	16	505	357	566	124	548	344	558	400	804	178
*deleted*	5,212	545	7,719	5,850	72,626	26	11,762	5,565	10,521	396	7,224	5,613	6,782	6,375	7,601	2,400
*exploit*	2,759	241	3,749	2,273	15,859	23	3,322	1,594	3,009	264	2,403	1,524	2,313	1,812	2,944	793
*oracle*	3,210	350	5,233	4,423	27,466	24	6,852	4,905	8,131	363	5,629	4,910	5,509	5,346	5,737	1,668
*policy*	2,134	126	2,792	1,558	18,161	21	1,716	1,044	1,740	247	1,437	1,011	1,428	1,145	1,794	440
*spyware*	12,035	2,287	19,470	14,186	N/A	N/A	N/A	N/A	36,902	441	23,759	18,126	21,863	20,935	22,469	8,079
*web-client*	14,884	1,685	23,253	19,953	N/A	N/A	N/A	N/A	59,063	776	65,057	61,183	64,150	63,158	64,793	15,216

^1^TCAM emulation.

^2^Pipelined TCAM emulation with a pipelined priority encoder in [21].

^3^DFA-based string matching in [16] with binary state encoding.

^4^DFA-based string matching in [16] with one-hot state encoding.

^5^Pipelined NFA-based string matching in [26] without pre-decoding scheme, pipelined priority encoder, and merged state transitions.

^6^Pipelined NFA-based string matching in [19] with pre-decoding scheme and without pipelined priority encoder &merged state transitions.

^7^Pipelined NFA-based string matching with pre-decoding scheme & pipelined priority encoder and without merged state transitions.

^8^Number of used LUT & FF pairs.

^9^Number of fully used LUT & FF pairs.

## Conclusion

This paper proposes a pipelined NFA-based string matching scheme with a new technique called merged state transitions. In addition, the pipelined priority encoder is adopted in order to maximize the operating frequency. The proposed string matching scheme is evaluated based on realistic experimental environments using the automatically generated RTL code, commercial synthesis tool, and state-of-the-art FPGA. Experimental data shows that the proposed string matching scheme can reduce the number of LUTs greatly and achieve high throughput per one character input up to 75.5% and 5.56 Gbps, respectively. As shown in [28, 29], because the pipelined NFA-based string matching scheme can process multiple chunks of characters in parallel, it is expected that throughput can be enhanced by equipping multiple instances. Therefore, the proposed string matching scheme can be extended. Considering the conceptual idea and experimental data, it is concluded that the proposed pipelined string matching scheme can be helpful to achieve high performance with low hardware cost.
